# Management of radiation therapy‐induced vaginal adhesions and stenosis: A New Zealand survey of current practice

**DOI:** 10.1002/jmrs.386

**Published:** 2020-04-08

**Authors:** Janeane Summerfield, Aidan Leong

**Affiliations:** ^1^ Department of Radiation Therapy Wellington Blood and Cancer Centre Wellington New Zealand; ^2^ Department of Radiation Therapy University of Otago Wellington New Zealand; ^3^ Bowen Icon Cancer Centre Wellington New Zealand

**Keywords:** side effects, patient care, gynaecology, radiation therapy, pelvis, dilation, stenosis

## Abstract

**Introduction:**

Vaginal dilation is often indicated as an intervention for the management of radiation therapy‐induced vaginal adhesions and stenosis (RTVAS). However, limited research exists underpinning this intervention and diversity in patient recommendations internationally are reported. In the absence of New Zealand (NZ) national guidelines regarding the management of RTVAS, a survey of NZ radiation therapy departments was conducted to gain an overview of current practice.

**Methods:**

A two‐section online survey was developed to capture RTVAS education and management overview across NZ. Section one focused on departmental resourcing and section two on local standard practice regarding vaginal dilator usage. One RTVAS education representative from each department was invited to complete the survey.

**Results:**

Eight of nine NZ departments completed the survey. Consistent treatment indications were identified for RTVAS patient education with the involvement of diverse staffing groups at various time‐points throughout the treatment process. Protocols for RTVAS management existed in each RT department with staff commonly trained by informal peer observation. Dilator usage was recommended regardless of patient sexual activity. Agreement was shown regarding the recommended start time of dilator usage and frequency. The recommended duration of dilator use post‐treatment varied from 6 months to greater than 36 months.

**Conclusions:**

This work illustrates both concordance and diversity in practice and contributes to the limited body of literature available. Further research is warranted to explore patterns of practice between departmental protocols and individual practitioners in further detail.

## Graphical abstract

How do New Zealand's RT departments manage RT‐induced vaginal adhesions and stenosis for female pelvic cancer patients? For the first time, the results of a national survey are published and give an overview of current practice in New Zealand.

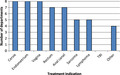

## Introduction

Radiation therapy (RT) is commonly used to treat pelvic tumours including cervical, endometrial, vaginal and anorectal malignancies. Female patients who receive RT for pelvic cancers may experience side effects such as adhesions, stenosis and shortening of the vagina due to fibrosis.^1^ These physical changes can result in long‐term sexual dysfunction for women, as well as compromising follow‐up pelvic examinations^2^, ^3^ and potentially surveillance of disease recurrence.^4^ Dilation is often indicated as an intervention for the management of adhesions and stenosis.^5^ This involves inserting a phallic shaped object into the vagina at regular intervals to open and stretch the vaginal tissues. Dilators are usually manufactured in graduated sizes from rigid plastic or silicone.^5^

A 2014 Cochrane review^5^ found studies of limited quality with conflicting outcomes regarding the benefit of vaginal dilator use. The use of vaginal dilators during RT was not supported, while their use after RT was indicated, provided acute vaginal side effects had resolved. Furthermore, these authors identified challenges in conducting more robust research in the future, given the intimate and ethically complex nature of the dilator intervention.

Despite this, vaginal dilation remains an internationally accepted management strategy for radiation therapy‐induced vaginal adhesions and stenosis (RTVAS) with vaginal dilation reported as a standard intervention procedure in the United Kingdom (UK), Netherlands and Australia.^1^, ^2^, ^4^, ^6^, ^7^, ^8^, ^9^ Patient education is a fundamental component of RTVAS side effect management. However, education and guidelines vary including appropriate time interval to commence vaginal dilation, frequency and durations of use, size of dilator and technique.^1^, ^2^, ^6^, ^8^, ^9^ In the absence of New Zealand (NZ) national standards for the management of RTVAS, the diversity in clinical practice is uncertain. The purpose of this survey was to establish an overview of the management of RTVAS within NZ RT departments.

## Methods

A ten‐question online survey (Supplementary Table 1) was developed by the authors to capture an overview of the standard education provided to female pelvic patients relating to RTVAS across all nine RT departments in NZ.^11^ Questions were aligned with previously published survey results from the UK^8^ and Australia^9^ for comparability. The first five‐question section of the survey concerned departmental resourcing. Participants were asked to identify which patient groups receive education regarding RTVAS; the staffing group(s) that provide this education; the timing of education; and the training of staff that provide education. The second section of the survey asked for local practice regarding recommended vaginal dilator usage. This specifically included the time of commencement, frequency and duration of dilator use. All nine RT departments in New Zealand were invited to participate via email to a national RT educator contact list, with one representative directly involved in RTVAS patient education asked to complete the online survey. The survey was anonymous and did not request information that would directly identify the individual completing the survey, nor their clinical department. Data were collected using Survey Monkey and analysed electronically using Microsoft Excel. This study was approved by the University of Otago Human Ethics Committee (reference number D19/124).

## Results

The survey was conducted during a three‐week period of August 2018. Of the nine New Zealand departments invited to participate, eight (89%) completed the survey.

### Departmental resourcing

**Figure 1:**
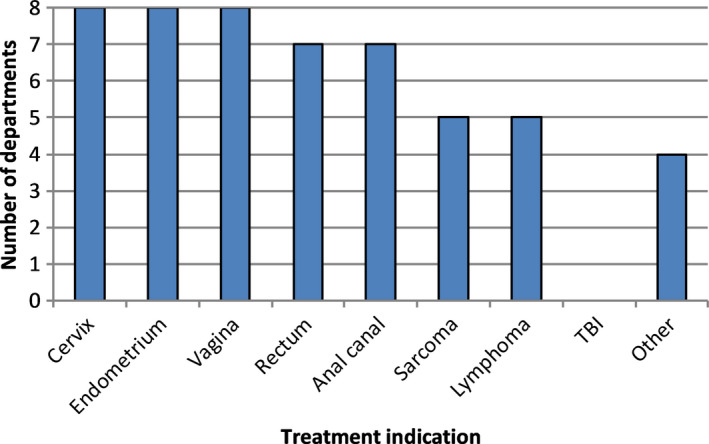
Departmental provision of RTVAS education by treatment indication. TBI = total body irradiation. Free text responses to ‘Other’ included any pelvic site receiving>30Gy, and any treatment involving the vagina.

All departments (100%) reported standardly educating female pelvic patients regarding the incidence and management of RTVAS. Figure 1 shows the treatment indications for which departments reported providing RTVAS education. Gynaecological diagnoses were consistently included (100%), as were those of the rectum (87.5%) and anal canal (87.5%). Additional indications reported as ‘other’ included any pelvic site receiving more than 30Gy and any treatment involving the vagina.

**Figure 2:**
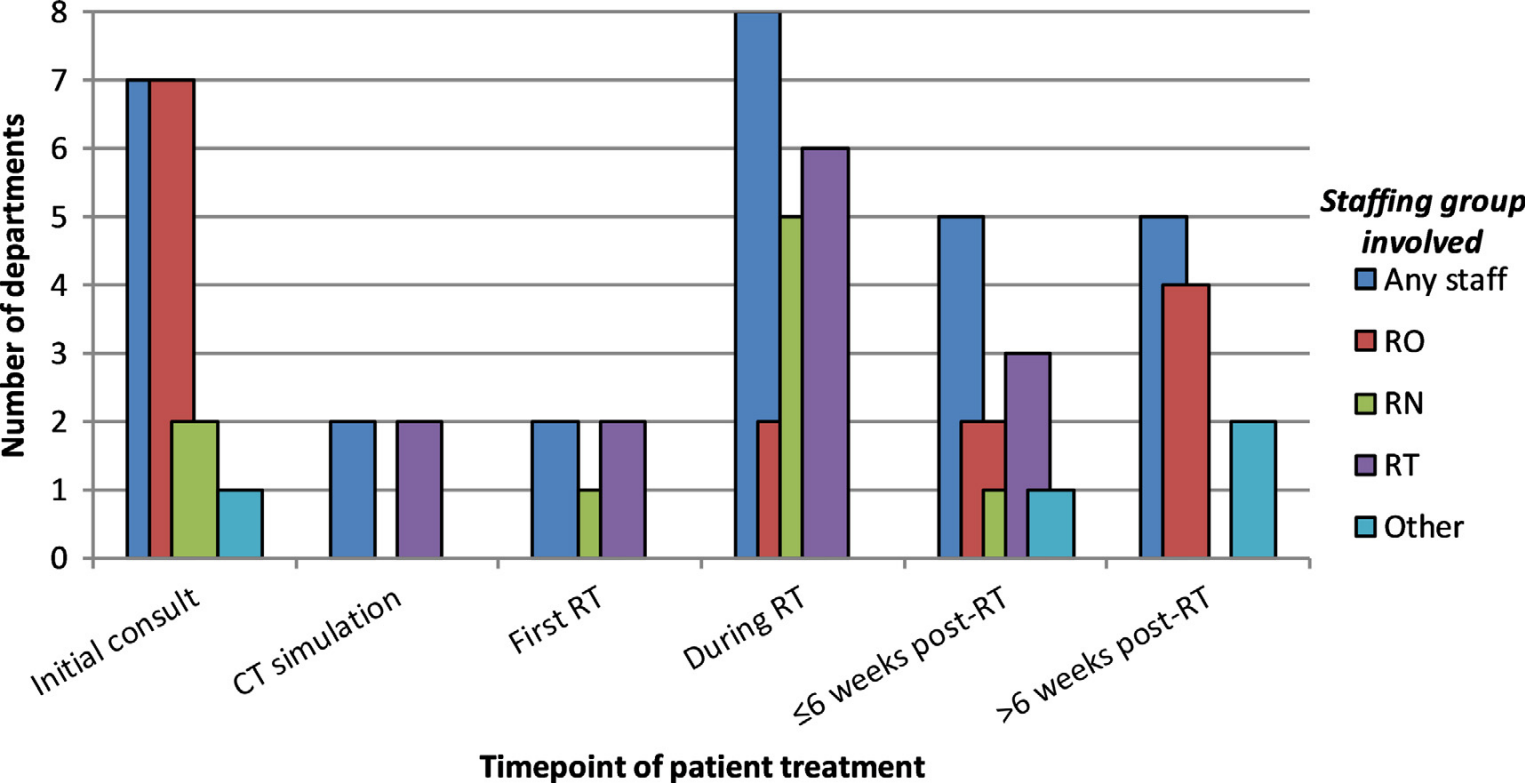
Staffing groups involved in the provision of RTVAS education and management. ‘Any staff’ denotes the number of departments reporting the provision of RTVAS education and management by any staffing group at each timepoint, irrespective of speciality. RO = Radiation Oncologist, RN = Registered Nurse, RT = Radiation Therapist. The sum of individual staff groups may exceed the ‘any staff’ value as multiple specialities were jointly involved at a particular time point for some departments. Free text responses to ‘Other’ included gynaecology clinical nurse specialist.

Staffing groups involved in the provision of RTVAS education and management was seen to vary across NZ departments dependent on the time point of the patient’s treatment journey (Fig. 2). At a patient’s initial consultation, radiation oncologists were reported as involved in providing education in all but one department (87.5%). Education was only provided by two departments (25%) at either planning CT or a patient’s initial treatment appointment. During the course of treatment, both radiation therapists (75%) and oncology nurses (62.5%) commonly provided RTVAS support. This support was less frequently reported within the first 6 weeks of completion of treatment but when it was provided, it was by radiation therapists most commonly (37.5%). Beyond 6 weeks post‐treatment, four departments (50%) reported the involvement of a radiation oncologist in education or management of RTVAS. The ‘other’ reported staffing group was identified as gynaecology clinical nurse specialists (gynae CNS).

Only one of eight departments (12.5%) reported the standard referral of female pelvic patients to a specialist service for the management of RTVAS (gynae CNS). However, this was commented upon by several other survey respondents that a specific referral would be offered on an ad hoc basis.

All respondents (100%) reported a departmental guideline or protocol for the purposes of training staff in the education and management of RTVAS. Additionally, seven departments (87.5%) utilised informal ‘on‐the‐job’ observation and training, while three (35%) had a formal departmental training programme. Other resources employed included pelvic floor physiotherapy education, patient information booklets and online e‐learning modules.

### Vaginal dilator recommendations

**Figure 3:**
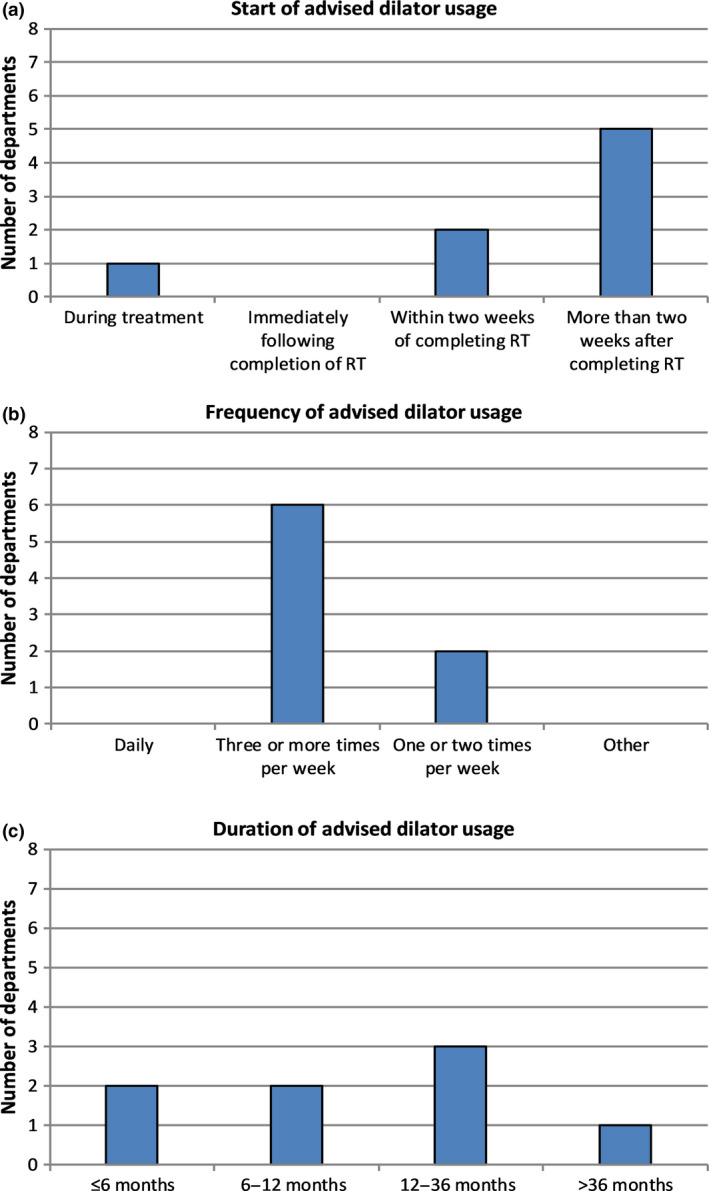
Distribution of departmental responses regarding the time of commencement (A), frequency (B), and duration (C) of vaginal dilator usage.

Eight NZ departments reported that all patients, irrespective of sexual activity, were standardly provided with vaginal dilators and were recommended to use lubricant. Most departments (87.5%) advised patients to begin using a dilator after the completion of treatment, either within two weeks or after more than two weeks (Fig. 3A). One department advised patients to begin dilation during treatment with instructions to cease once any RT side effects manifested. Dilators were most commonly (75%) recommended to be used 3 or more times per week (Fig. 3B). The duration for which dilators were recommended to be used varied across departments (Fig. 3C). Responses ranged between less than 6 to more than 36 months.

## DISCUSSION

To the authors’ knowledge, this is the first published survey of contemporary NZ practice in the management of RTVAS. This is particularly relevant in the absence of a national standard or consensus guideline. The results show clear areas of alignment in practice, as well as certain areas of diversity.

When interpreting the findings of this study, the limitations of the staff survey utilised should be recognised. Because only a single representative of each department completed the survey, responses may have been biased by the perceptions of the respondent and thus not represent true practice within each department’s wider multidisciplinary team. A broader national survey of individual clinicians was beyond the scope of this work, but would be worthy of future investigation. The structure of the survey was designed by the authors based on their clinical and educational expertise as well as alignment with previously published survey results in the field. This was considered appropriate for the aim of the study; however, future work investigating RTVAS education and management in further detail would benefit from a more formalised survey design process including representation of the wider multidisciplinary team.

Our survey showed general agreement between NZ departments for pelvic treatment sites where education is provided regarding the incidence and management of RTVAS. In 2006, a survey of UK practitioners demonstrated comparatively more divergence in the patient cohorts targeted.^8^ Of particular note, non‐gynaecological pelvic diagnoses such as rectal and anal canal cancer were only recommended dilator management by 22% of respondents.^8^ In comparison, seven of eight NZ departments standardly recommended RTVAS management for both rectal and anal canal diagnoses. These sites are less commonly discussed in the wider literature, though it has been reported that more than two thirds of women treated with RT for rectal or anal cancer experience some degree of RTVAS.^10^ Two respondents in the NZ survey also reported a broader approach, providing RTVAS education to all female pelvic patients treated to 30Gy or above.

The literature regarding the provision of RTVAS education has predominately focussed on the role of gynaecology or oncology nurses.^2^, ^7^, ^9^, ^11^, ^12^ This NZ survey showed an interdisciplinary approach to RTVAS management and dilator education for patients, with a variety of RO, RN, gynaecology CNS and RT involvement at different time points in patients’ treatment journeys.

Staff training for RTVAS management was carried out in all responding departments with the use of a protocol or guideline, which was commonly supported by informal on‐the‐job training. A critical analysis of departmental protocols presents an opportunity for further work investigating national practice patterns in more detail.

Several studies have reported patient sexual activity as an influencing factor in the recommendation of vaginal dilator usage for the management of RTVAS.^2^, ^8^, ^9^ In the UK, 20% of survey respondents stated patient sexual frequency as a factor in offering patients vaginal dilation education.^8^ Lancaster^9^ found that sexual activity could be both an indication and contraindication for dilator recommendation between Australian departments. In contrast, all responding NZ departments in the current survey stated that female pelvic patients’ were provided with vaginal dilators, irrespective of sexual activity.

Consistent with international practice, all survey respondents recommended their female pelvic patients use lubricant with dilators.^2^, ^6^, ^8^ Of note, recent research has indicated that lubricants commonly used in the clinical environment may not the most suitable for long‐term patient use.^13^ This is partly due to the preservatives in some lubricants which may be detrimental to the vaginal microbiome. Disruptions to microbiome levels may contribute to bacterial vaginosis, contact dermatitis and *Candida albicans* growth.^13^ This is an important consideration given the prolonged time frames that female pelvic patients’ may be advised to use lubricant and dilators.^2^, ^4^, ^5^, ^6^, ^7^, ^8^, ^9^, ^10^

Areas of diversity in this survey included the time at which patients are advised to start using vaginal dilators. Similarly, the advice given regarding the duration of dilator usage post‐treatment also varied considerably from <6 months to ≥36 months. This result was similar to UK and Australian surveys where no clear consensus was demonstrated across respondents.^8^, ^9^ Of note, only one NZ department advised patients to continue using dilators ≥36 months post‐treatment. Comparatively, 41% and 60% of UK and Australian respondents, respectively, recommended indefinite use of vaginal dilatation.^8^, ^9^

## Conclusion

This study establishes a current overview of NZ departmental practice regarding the management of RTVAS. Though areas of both concordance and diversity in practice have been shown, this work contributes to the limited body of literature available in the field. The lack of evidence guiding RTVAS management is widely acknowledged, as are the challenges that continue to hinder further research. This is due to the subjective, intimate and ethically complex nature of investigating patient RTVAS experiences.^5^ However, further research is warranted to explore patterns of practice between departmental protocols and individual practitioners in more detail. The findings of this survey form a preliminary reference for national RTVAS management and ongoing practice development.

## Supporting information


**Table S1.** Online survey questions and answer options for respondents.

## References

[1] Morris L , Do V , Chard J , Brand AH . Radiation‐induced vaginal stenosis: current perspectives. Int J Women’s Health 2017; 9: 273–279. Published online. May 2, 2017. 28496367 10.2147/IJWH.S106796 PMC5422455

[2] Bakker RM , ter Kuile MM , Vermeer WM , et al. Sexual rehabilitation after pelvic radiotherapy and vaginal dilator use: consensus using the Delphi method. Int J Gynecol Cancer 2014; 24(8): 1499–506. 25248115 10.1097/IGC.0000000000000253

[3] Law E , Kelvin J , Thom B , et al. Prospective study of vaginal dilator use adherence and efficacy following radiotherapy. Radiother Oncol 2015; 116: 149–155. 26164775 10.1016/j.radonc.2015.06.018 PMC5028178

[4] Damast S , Jeffery D , Son CH , et al. Literature review of vaginal stenosis and dilator use in Radiation Oncology. Pract Radiat Oncol 2019; 7: 73–79. 10.1016/j.prro.2019.07.001 PMC7944435 31302301

[5] Miles T , Johnson N . Vaginal dilator therapy for women receiving pelvic radiotherapy. Cochrane DB Syst Rev 2014; (9): CD007291. 10.1002/14651858.CD007291.pub3 PMC6513398 25198150

[6] Cancer institute NSW . eviQ. Supporting document – management of radiation induced vaginal stenosis. 2016 Available from https://www.eviq.org.au/clinical‐resources/radiation‐oncology/1867‐management‐of‐radiation‐induced‐vaginal‐steno. Accessed 07 Sept 2018.

[7] International guidelines on vaginal dilation after pelvic radiotherapy . Produced by the International Clinical Guideline Group, chaired by Dr Tracie Miles, President, National Forum of Gynaecological Oncology Nurses, UK Available from https://owenmumford.com/us/wp‐content/uploads/sites/3/2014/11/Dilator‐Best‐Practice‐Guidelines.pdf Accessed 03 Jan 2020.

[8] White ID , Faithfull S . Vaginal dilation associated with pelvic radiotherapy: A UK survey of current practice. Int J Gynecol Cancer 2006; 16(3): 1140–6. 16803497 10.1111/j.1525-1438.2006.00452.x

[9] Lancaster L . Preventing vaginal stenosis after brachytherapy for gynaecological cancer: an overview of Australian practices. Eur J Oncol Nurs 2004; 8(1): 30–39. 15003742 10.1016/S1462-3889(03)00059-0

[10] Son C , Law E , Hun J , et al. Dosimetric predictors of radiation‐induced vaginal stenosis after pelvic radiation therapy for rectal and anal Cancer. Int J Radiation Oncology Biol Phys 2015; 92(3): 548–554. 10.1016/j.ijrobp.2015.02.029 PMC4822494 25936810

[11] The National Radiation Oncology Plan 2017–2021. New Zealand Government, Ministry of Health. 2017. Available from https://www.health.govt.nz/system/files/documents/publications/national‐radiation‐oncology‐plan‐may17.pdf Accessed 03 Jan 2020.

[12] Bergin B , Hocking A , Robinson T , Kabel D . Continuing variation and barriers to nurse‐led vaginal dilator education for women with gynaecological cancer receiving radiotherapy. Eur J Oncol Nurs 2016; 24: 20–21. 27697273 10.1016/j.ejon.2016.08.001

[13] Edwards D , Penay N . Treating vulvovaginal atrophy/genitourinary syndrome of menopause: How important is vaginal lubricant and moisturizer composition? Climacteric 2016; 19(2): 151–161. 26707589 10.3109/13697137.2015.1124259 PMC4819835

