# Supplementary material for: Management of radiation therapy‐induced vaginal adhesions and stenosis: A New Zealand survey of current practice
Source: J Med Radiat Sci. 2020 Apr 8;67(2):128–33. doi: 10.1002/jmrs.386 (PMC7276182; doi:10.1002/jmrs.386)
Supplement: Supplementary file 1 — Table S1. Online survey questions and answer options for respondents. [file JMRS-67-128-s001.docx]

*Supplementary Table 1. Online survey questions and answer options for respondents.*

|  | **Question** | **Answer options for respondents** |
| --- | --- | --- |
|  | *Section 1: Departmental Resourcing* | |
| 1 | Within your department, are female pelvic patients’ standardly educated regarding the incidence and management of RTVAS? | - Yes - No |
| 2 | For which treatment indication(s) is education provided regarding the incidence and management of RTVAS? (select all that apply) | - Cervix - Endometrium - Vagina - Rectum - Anal canal - Sarcoma (pelvic) - Lymphoma (pelvic) - Total body irradiation - Other (please specify). |
| 3 | Which staffing group(s) are standardly involved in the provision of education and management relating to RTVAS? (select all that apply) | *Staffing group options*   - Radiation therapist - Oncology nurse - Radiation oncologist - Other staffing group (please specify)   *Time point options for each staffing group*   - Initial consultation for RT - Planning CT appointment - First RT treatment - During RT - ≤6 weeks after completing RT - >6 weeks after completing RT |
| 4 | Following completion of RT, are female pelvic patients standardly referred to any service specifically regarding the management of RTVAS and/or sexual function? | - Yes (please specify) - No |
| 5 | What resources are used to train staff involved in the provision of education and management relating to RTVAS? | - Formal departmental training programme - Informal ‘on-the-job’ observation/teaching. - Departmental protocol/guideline. - Other resources (please specify). |
|  | *Section 2: Vaginal dilator usage* | |
| 6 | Which groups of female pelvic patients’ are standardly provided with vaginal dilators? (select all that apply) | - All patients irrespective of sexual activity - Patients who **are** currently sexually active - Patients who **are not** currently sexually active, but may wish to in the future. - Patients who **are not** currently sexually active and do not anticipate being sexually active in the future. - Please state the brand of vaginal dilators provided |
| 7 | Is lubricant recommended for female pelvic patients using dilators? | - Yes (please specify brand) - No |
| 8 | When are patients advised to begin using dilators? | - During treatment. - Immediately following completion of RT - Within two weeks of completing RT - More than two weeks after completing RT |
| 9 | How frequently are patients advised to use dilators? | - Daily - One or two times per week - Three or more times per week - Other (please specify) |
| 10 | How long are patients advised to continue using dilators after completion of RT? | - ≤6 months - 6-12 months - 12-36 months - >36 months - Please state any specific criteria used to recommend when patients should discontinue using dilators |
